# Solitary gastric Peutz-Jeghers polyp: a case report

**DOI:** 10.11604/pamj.2022.41.65.29526

**Published:** 2022-01-24

**Authors:** Amal Khsiba, Samir Bradai, Abdelawaheb Nakhli, Emna Chelbi, Moufida Mahmoudi, Asma Ben Mohamed, Mouna Medhioub, Lamine Hamzaoui, Mohamed Mousadek Azouz

**Affiliations:** 1 Department of Gastroenterology, Mohamed Tahar Maamouri Hospital, Nabeul, Tunisia,; 2 Department Histopathology, Mohamed Tahar Maamouri Hospital, Nabeul, Tunisia

**Keywords:** Peutz-Jeghers syndrome, rectum, cancer, pancreatitis, case report

## Abstract

Peutz-Jeghers syndrome is an inherited condition that is characterized by mucocutaneous pigmentation and hamartomatous polyposis in the gastrointestinal tract. It increases significantly the risk for developing of several cancers such as breast, colon, rectum, pancreas and stomach. Solitary Peutz-Jeghers polyp is defined as a unique hamartomatous polyp having the same histological features as Peutz-Jeghers syndrome polyps without associated intestinal polyposis, mucocutaneous pigmentation and family history of Peutz-Jeghers syndrome. Gastric solitary Peutz-Jeghers polyp is extremely rare. We found only 13 cases in the literature. We report a new case of solitary gastric Peutz-Jeghers polyp associated with a branch duct intraductal papillary mucinous neoplasm revealed by an acute pancreatitis. Computed tomography of the abdomen found a branch duct intraductal papillary mucinous neoplasm with a pedicled polypoid formation in the greater gastric curvature. Endoscopic resection was performed without complications. Histologic examination showed Peutz-Jeghers hamartomatous polyp. The risk of cancer remains unclear in this entity. Therefore, the follow-up of these patients is necessary because of the possible risk of malignancy.

## Introduction

Peutz-Jeghers syndrome (PJS) is a rare autosomal dominant genetic disease due to a germline mutation in STK11 gene [1]. It is characterized by hamartomatous gastrointestinal polyposis and mucocutaneous pigmentation [1]. Rare cases of solitary or sporadic Peutz-Jeghers polyp (SPJP) have been reported in the literature. Solitary or sporadic Peutz-Jeghers polyp is defined as a unique hamartomatous polyp having the same histological features as Peutz Jeghers syndrome polyps without associated mucocutaneous pigmentation or family history of Peutz-Jeghers syndrome [2]. The SPJP occurs predominantly in the small bowel [3]. Gastric location is extremely rare. We present a case of gastric SPJP treated by endoscopy along with a review of the literature.

## Patient and observation

**Patient information:** a 81-year-old man with a history of high blood pressure presented to the hospital with intense epigastric pain radiating to the back associated with vomiting. The patient had no family history of polyps or tumors in the gastrointestinal tract.

**Clinical findings:** the clinical examination found a temperature of 37.8°, blood pressure at 130/80mmHg, a heart rate of 80 beats per minute and a tenderness of the abdomen with no other alterations in the examination (including in skin/appendages examination).

**Timeline of the current episode:** this was the first episode, and have never presented such clinical manifestations. The episode began 72 hours before his admission to the emergency room.

**Figure 1:**
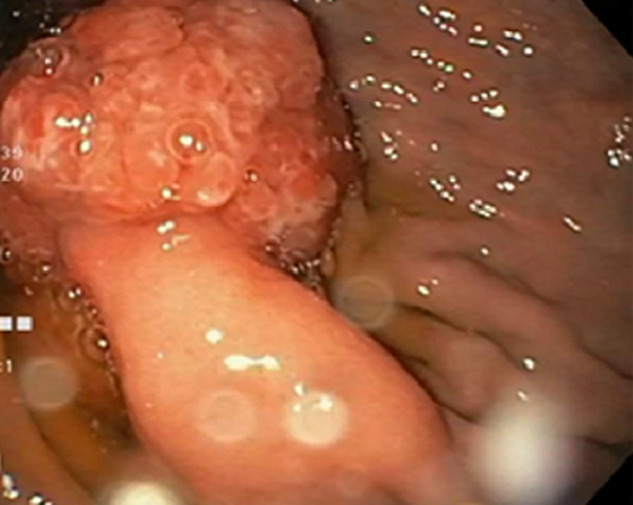
gastroscopy showed a pedunculated polyp measuring 40 mm in the greater curvature

**Figure 2:**
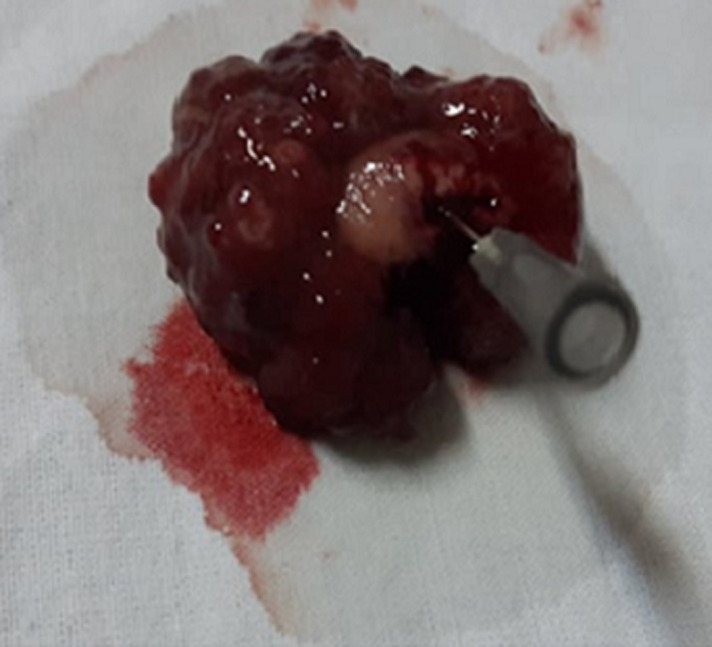
macroscopic appearance of the polyp after endoscopic resection

**Figure 3:**
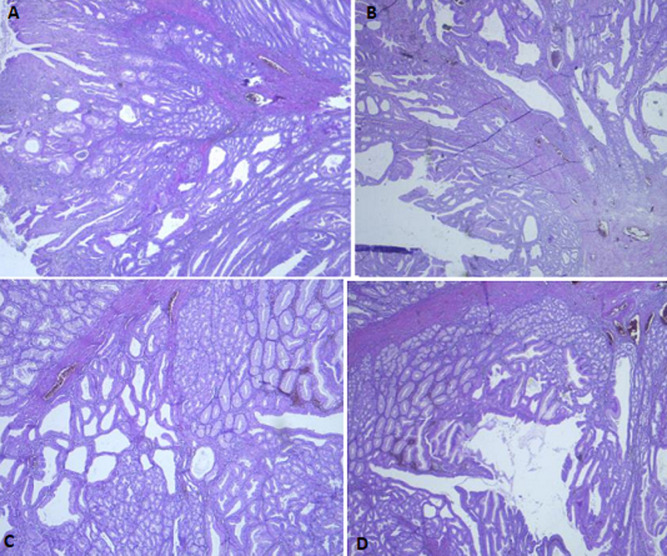
(A, B, C, D) histological examination of the polyp showed branching bundles of smooth muscle fibers from the muscularis mucosae extended to the polyp

**Figure 4:**
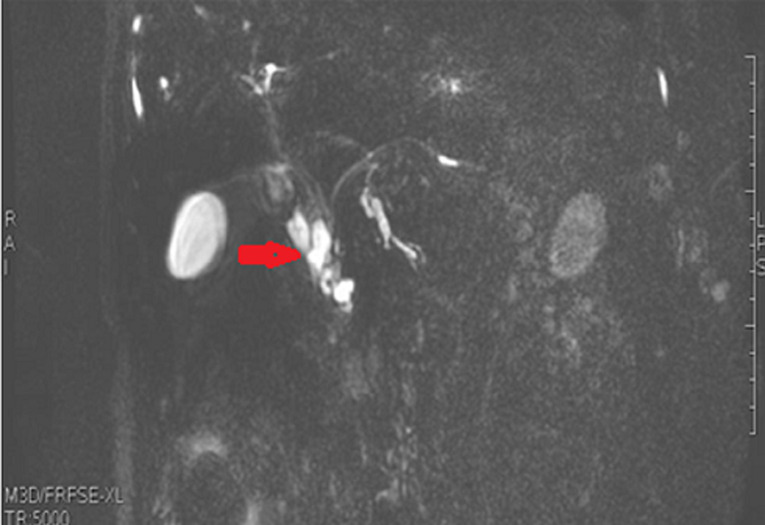
pancreatic magnetic resonance imaging showed branch duct intraductal papillary mucinous neoplasm of the head of the pancreas

**Diagnostic assessment:** the laboratory tests found a lipase level of 3500 U/L (range: 0-160 U/L) confirming the diagnosis of acute pancreatitis. The white blood cell count, C-reactive protein and liver function tests were normal. A Computed tomography (CT) scan of the abdomen was performed after 72 hours to assess the severity of the pancreatitis and look for a possible cause. The pancreas had a normal size and density with cystic formations communicating with the main pancreatic duct, evoking branch duct intraductal papillary mucinous neoplasm (IPMN). The CT scan found fortuitously a pedicled polypoid formation with endoluminal development located in the greater gastric curvature, measuring 40×20mm. We completed with esophagogastroduodenoscopy that revealed a pedunculated polyp measuring 40mm with ulcerated surface and large pedicle, located on the greater curvature (Figure 1). Endoscopic resection was performed without complications (Figure 2). Histological examination showed a hamartomatous polyp with branching bundles of smooth muscle fibers from the muscularis mucosae extended to the polyp which is covered by normal mucosa, confirming the diagnosis of Peutz-Jeghers hamartomatous polyp (Figure 3). No polyps were found on ileocolonoscopy. Pancreatic Magnetic Resonance Imaging (MRI) was carried out to better characterize the pancreatic cystic formations. It showed multiple cystic formations of the head of the pancreas communicating with the main pancreatic duct, allowing to confirm the diagnosis of branch duct intraductal papillary mucinous neoplasm (Figure 4).

**Diagnosis:** our patient had no mucocutaneous pigmentation, gastrointestinal polyposis or family history of PJS. Therefore, the diagnosis of a SPJP was retained. Our gastric SPJP is associated with a branch duct intraductal papillary mucinous neoplasm.

**Therapeutic intervention:** the patient underwent a hot snare polypectomy with a saline injection of the stalk. Concerning the intraductal papillary mucinous neoplasm, we opted for a follow-up because of the advanced age of the patient.

**Follow-up and outcome of interventions:** the patient has been followed up regularly and he is currently doing well, one year after the diagnosis.

**Patient perspective:** he was satisfied with the diagnostic and the proposed care.

**Patient consent:** he has given his consent for his images and other clinical information to be reported in the journal. The patient understands that his name and initials will not be published.

## Discussion

Sporadic Peutz-Jeghers polyp have been reported in few cases in the literature. They are located predominantly in the small intestine, followed by the colorectal region [3]. The gastric location is extremely rare. A gastric SPJP was described for the first time by Kuwano *et al*. in 1989 [4]. In the English literature, only 13 cases of gastric SPJP were reported (Table 1) [3,5,6]. The STK11 gene mutation is considered as the cause of the PJS. It is found in 90% of patients [7]. In SPJP, the genetic analysis was performed in three cases [3]. It was found negative for STK11 mutation in all cases. Therefore, SPJP is probably a different clinical entity [3]. The median age of patients with gastric SPJP at diagnosis was 50 years [3,5,6]. Our patient is the oldest of all cases. The median age at diagnosis is higher than that of patients with PJS [2]. Clinical manifestations are non-specific including abdominal discomfort, abdominal pain, gastrointestinal bleeding or anemia [3,5,6]. The size of gastric SPJP ranges from 5 to 150mm [3,5,6]. Histology shows the same features as PJS polyps with a distinctive arborization within the lamina propria of smooth muscle bundles, arising from muscularis mucosae [2]. Some authors have suggested that the solitary gastric Peutz Jeghers polyps have less branching of the muscularis mucosae as compared with familial PJS polyps [4].

**Table 1:** different cases of solitary gastric Peutz-Jeghers polyp reported in the literature

Authors	Year	age	Gender	Symptoms	Size (mm)	Treatement
Kuwano *et al*.	1989	17	Man	Epigastralgia with diarrhea	20	Endoscopy
Grisendi *et al*.	1990	53	Woman	N/A	20	Endoscopy
Hunt *et al*.	1996	27	Woman	Vomiting, weight loss, melena	80	Surgery
Sakadamis *et al*.	2001	47	Woman	Epigastralgia, nausea, melena	75x50	Surgery
O'Loughlin *et al*.	2002	38	Woman	Epigastric discomfort, abdominal fullness, nausea, regurgitation	70x40	Endoscopy
Oncel *et al*.	2003	78	Man	Dyspepsia	5	Endoscopy
Harbaum *et al*.	2009	61	Man	N/A	10	Endoscopy
Jin *et al*.	2012	71	Woman	Epigastralgia, weight loss	40x30	Surgery
Lunca *et al*.	2014	43	Woman	Bleeding, abdominal discomfort,weight and appetite loss	150x70x50	Surgery
Shi *et al*.	2014	67	man	Abdominal pain and distension	25	Endoscopy
Yoshizawa *et al*.	2016	37	Woman	Epigastric discomfort	65x60x30	surgery
Bai-Cang Zou *et al*.	2017	53	Man	Left upper abdominal pain	110x80	Endoscopy
Goto *et al*.	2020	32	Woman	Epigastric pain	10	N/A
Present case	2021	81	Man	Acute pancreatitis	40	Endoscopy

N/A : non available

The treatment of SPJP is based on surgical or endoscopic resection, depending on the size and the location of the polyp. For the gastric location, polyps were resected endoscopically in seven cases and five patients underwent a gastric resection [3,5,6]. Peutz-Jeghers syndrome increases significantly the risk of cancers of the digestive tract and other organs essentially pancreas, colon, stomach, breast, ovary, testis, uterus, cervix and lung [1]. Whether SPJP increases cancer risk remains controversial. Oncel *et al*. followed up 8 patients with SPJP (5 in the colon, 2 in the duodenum, 1 in the stomach) for a period of 11.5 years [8]. No cases of PJS associated cancers were found. However, some authors reported cases of malignancies associated with SPJP (pancreas, ovary, aglomus tympanicum tumor, prostate, rectum, lung, liver, thyroid) [9,10] or malignant components in the polyps [10-13]. None of the 13 gastric SPJP reviewed in the literature had malignant components in the polyp. Only one case of gastric SPJP with a high-grade tubular adenoma in the colon was reported [7]. In our case, the polyp was associated with intraductal papillary mucinous neoplasm which is a precancerous lesion of pancreatic adenocarcinoma. Due to the small number of cases reported, the cancer risk of SPJP is not yet clear. Considering that there are some cases of solitary Peutz-Jeghers hamartomatous polyp with malignant components or associated with extraintestinal malignancies, we propose the follow-up of these patients because of the possible risk of malignancy.

## Conclusion

Solitary gastric Peutz-Jeghers polyps are very rare with only 13 cases described in the literature. Our case is associated with intraductal papillary mucinous neoplasm (IPMN) which is a precancerous lesion of pancreatic adenocarcinoma. The risk of cancer remains unclear in this entity. Therefore, the follow-up of these patients is necessary because of the possible risk of malignancy. A long-term follow-up of patients with SPJP can provide new informations to better understand this disease.
